# Heme Oxygenase-1 in Gastrointestinal Tract Health and Disease

**DOI:** 10.3390/antiox9121214

**Published:** 2020-12-02

**Authors:** Jose D. Puentes-Pardo, Sara Moreno-SanJuan, Ángel Carazo, Josefa León

**Affiliations:** 1 Research Unit, Instituto de Investigacion Biosanitaria de Granada, ibs.GRANADA, 18012 Granada, Spain; 2 Department of Pharmacology, Faculty of Pharmacy, University of Granada, 18011 Granada, Spain; 3 Cytometry and Microscopy Research Service, Instituto de Investigacion Biosanitaria de Granada, ibs.GRANADA, 18012 Granada, Spain; sara.moreno@ibsgranada.es; 4 Genomic Research Service, Instituto de Investigacion Biosanitaria de Granada, ibs.GRANADA, 18012 Granada, Spain; angelcarazogallego@gmail.com; 5 Clinical Management Unit of Digestive Disease, San Cecilio University Hospital, 18016 Granada, Spain

**Keywords:** heme oxygenase, gastrointestinal tract, cancer, diabetes, pancreatitis, inflammatory bowel disease, peptic ulcer disease, fatty liver disease, ferroptosis

## Abstract

Heme oxygenase 1 (HO-1) is the rate-limiting enzyme of heme oxidative degradation, generating carbon monoxide (CO), free iron, and biliverdin. HO-1, a stress inducible enzyme, is considered as an anti-oxidative and cytoprotective agent. As many studies suggest, HO-1 is highly expressed in the gastrointestinal tract where it is involved in the response to inflammatory processes, which may lead to several diseases such as pancreatitis, diabetes, fatty liver disease, inflammatory bowel disease, and cancer. In this review, we highlight the pivotal role of HO-1 and its downstream effectors in the development of disorders and their beneficial effects on the maintenance of the gastrointestinal tract health. We also examine clinical trials involving the therapeutic targets derived from HO-1 system for the most common diseases of the digestive system.

## 1. Introduction

Heme oxygenase (HO) is the enzyme that catalyzes the first and the rate-limiting step of heme oxidative degradation. During this reaction, HO requires NADPH and oxygen to degrade heme, releasing carbon monoxide (CO), free iron (Fe^2+^), and biliverdin, which is reduced to bilirubin by biliverdin reductase consuming another NADPH molecule, as by-products [1,2]. 

Three different isoforms have been described to the date, heme oxygenase 1 (HO-1), which is an inducible form in response to diverse chemical and physical stimuli; heme oxygenase 2 (HO-2) which is the constitutive form expressed throughout the body, but specifically in testes, endothelial cells and brain; and heme oxygenase 3 (HO-3), a poor catalytically-active form only found in rats [3,4]. Bacterial HO-like proteins have also been described, pointing out the evolutionary conserved activity of this enzyme through evolution, and has been postulated that gut microbiota may take part somehow in heme metabolism [5].

Despite of the existence of two active isoforms, most of the research has been focus on HO-1, since HO-1 knock-out animals present a highly intrauterine death ratio and overall severe chronic inflammation, accompanied by a predisposition to oxidative stress and iron metabolism dysfunction [6,7,8,9]. Meanwhile, HO-2 knockout models remain fertile and show less severe disturbances, mainly localized to those tissues in which HO-2 is higher expressed, such as the nervous system [10]; or even being positive under certain conditions like intracerebral hemorrhage [11]. In humans, there are only two described cases of HO-1 deficiency: A 6-year-old boy who suffered from hemolytic anemia, growth retardation, leukocytosis, elevated heme in serum, low serum bilirubin levels, and iron depositions throughout the body; and a 15-year-old girl who shared similar symptoms including severe hemolysis, inflammation, nephritis, and premature death [12,13].

HO-1 is expressed at relatively-low basal levels in all mammalian tissues but is up-regulated under stress conditions. However, this inducible form occurs at high levels in spleen macrophages [14] and Kupffer cells in the liver [15], due to its role in senescent red blood cells clearance, as well as in hematopoietic stem cells in the bone marrow to control the levels of heme, which is an erythroid differentiation factor [16]. At the cellular level, HO is anchored to the endoplasmic reticulum through a C-terminal transmembrane region facing the cytosol [17], although some evidences have shown that HO-1 can be translocated to mitochondria in lung and gastric epithelial cells, where is associated with a protective role [18,19]. Intriguingly, nuclear localization of HO-1 has also been described. Under stress conditions, HO-1 may be translocated to the nucleus, where it exerts non-enzymatic functions, regulating its own expression, as well as transcriptional factors related to oxidative stress responses, which might be in turn associated with cancer progression [20]. 

HO-1 is considered as both a potent anti-oxidative and a cytoprotective agent. These activities are exerted, respectively, by two different but synergic mechanisms: (1) The removal of heme group, which is a potent oxidative agent leading to generation of reactive oxygen species (ROS) [21]; and (2) as a result of multiple mechanism underpinning by its by-products CO, biliverdin/bilirubin and labile iron, which is not a direct action of its enzymatic activity [16]. Indeed, HO-1 induction and/or impairment has been associated with a huge variety of diseases such as cancer [20], neurodegenerative, and cardiovascular diseases [22,23], among others.

Apart from the spleen and liver, HO-1 levels also seem to be constitutively expressed at the gastrointestinal system [24]. Since the digestive system is continuously exposed to a wide variety of stress conditions, the HO system seems to play an important part in gastrointestinal tract health and disease. In this review we summarize the essential role of HO-1 and its end-products for ensuring the gastrointestinal tract health and how its dysfunction lead to several disorders of the esophagus, stomach, pancreas, liver, and gut. We also examine the current ongoing clinical trials exploring the therapeutic targets derived from the HO-1 system for the most common gastrointestinal diseases.

## 2. Molecular Regulation of HO-1 Expression 

The regulation of HO-1 expression is mainly exerted at transcriptional level. HO-1 is encoded by the *HMOX1* gene located on chromosome 22q12.3. It has five exons, four introns and three regulatory regions, one proximal (−0.3 Kb) and two distals from the promoter region (E1 at −4 kb and E2 at −10 kb) [25,26]. These regulatory regions contain diverse transcriptional factor binding sites (hypoxia-inducible factor 1 (HIF-1), nuclear factor kappa B (NF-κB), activator protein (AP-1) binding sites), stress responses elements (StRE), metal response elements (MtRE), and heat shock consensus (HSE) sequences), which are involved in the tuning of the cellular redox state [16,27]. This variety of regulatory elements allows the transcription of *HMOX1* in response to a plethora of oxidative and inflammatory stimuli among which stand out its own substrate heme, heavy metals, radiations, ROS, growth factors, and cytokines [16]. Despite of the diversity of regulatory elements, the dominant sequence motif is the StRE, which behaves similar to Maf response element (MARE) and the antioxidant response element (ARE) [28,29]. In consequence, among the different transcriptional factors, the nuclear erythroid 2-related factor (Nrf2) and Bach1 exert a pivotal role in *HMOX1* regulation, activating or repressing, respectively, its transcription [29].

**Figure 1:**
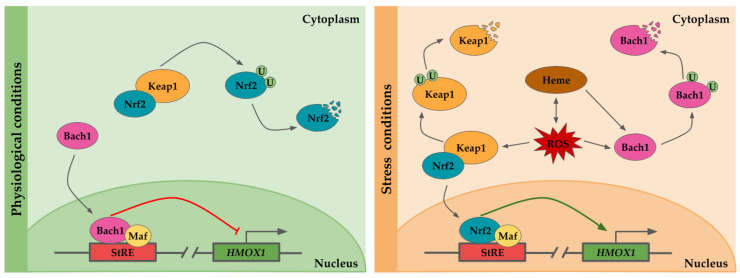
Scheme of the transcriptional regulation of *HMOX1*. The *HMOX1* gene regulatory region contains several stress response elements (StRE) to which transcription factors binds. Under physiological conditions, the nuclear erythroid 2-related factor (Nrf2) is sequestered in the nucleus by the Kelch-like ECH-associated protein (Keap-1), forming a heterodimer and targeting Nrf2 for ubiquitination and its subsequent proteosomal degradation. The *HMOX1* promoter is repressed by Bach1/Maf dimers bound to StRE. Heme is pro-oxidant and generates reactive oxygen species (ROS), which in turn may result in heme release from hemeproteins. In response to stress condition or presence of heme, bach1 translocation to the nucleus is inhibited, and it is targeted for degradation. Upon oxidative stress, Keap1 undergoes a conformational change which induces its ubiquitination and degradation, as well as Nrf2 translocation to the nucleus. Nrf2/Maf dimers transactivate *HMOX1* expression by binding to StREs.

Under physiological conditions Nrf2, a basic leucine zipper protein, is retained into the cytoplasm by the Kelch-like ECH-associated protein (Keap-1), which inhibits its translocation to the nucleus [30]. Keap-1 forms a heterodimer with Nrf2, sequestering and facilitating the Nrf2 targeting and ubiquitination by Cullin-RING E3 ubiquitin ligase complex for proteosomal degradation [30,31]. In parallel, Bach1 is translocated to the nucleus where it heterodimerizes to form a complex with small Maf protein, and then, the Bach1/small Maf complex binds to StRE, repressing HO-1 expression [32,33] (Figure 1). 

Upon oxidative conditions or elevated concentrations of heme, although under certain circumstances both situations might be equivalent since heme is a pro-oxidative molecule and oxidative stress may lead to heme release from hemeproteins [21], the repression exerted by Bach1 is relief [32]. Heme binds to Bach1 heme binding sites, inducing Bach1 conformational modifications. These changes prevent Bach1 binding to StREs and lead to Bach1 export from the nucleus, and its ubiquitination by E3 ubiquitin ligase HOIL-1 and proteosomal degradation [32,34]. In addition, oxidative stress may also release the HO-1 repression by Bach1 through oxidation of its sulfhydryl groups [35]. On the other hand, when the cells are subjected to oxidative stress, Keap1 undergoes a conformational change which results in Nrf2-Keap1 dissociation and Nrf2 translocation to the nucleus where it interacts with small Maf proteins, and binds to StRE, promoting *HMOX1* expression [30]. Prolonged exposition to oxidative stress results in Keap1 ubiquitination and proteosome-independent degradation [36] (Figure 1).

Several studies have revealed that nitric oxide (NO) stimulates HO-1 expression in different cell types [37,38]. This upregulation mediated by NO seems to occur through the activation of Nrf2/ARE complex, since mutations on ARE or Nrf2 negative mutants result in the abrogation of HO-1 induction by NO, at least in vascular smooth muscle cells [38]. NO exogenous or endogenous, produced by inducible NO synthase (iNOS) may display anti-inflammatory capacities, likely as a consequence of NO induction of HO-1 and the subsequent upregulation of the HO-1/CO pathway [39]. Curiously, the increase of HO-1 expression mediated by NO could not be just the result of the *HMOX1* transcription increase, but it also may be the result of an indirect HO-1 mRNA stabilization by NO [40]. 

Finally, and although is not a transcriptional regulation itself, the *HMOX1* expression also seems to depend on microsatellite dinucleotide repeat polymorphism (GT)_n_. In average, the size of GT repeat length ranges between 12 and 40 repeats. Short GT repeats (<25 repeats) have been associated with a robust *HMOX1* expression compared to larger repeats (>25) [41].

## 3. Cytoprotective and Deleterious Effects Mediated by HO-1

**Figure 2:**
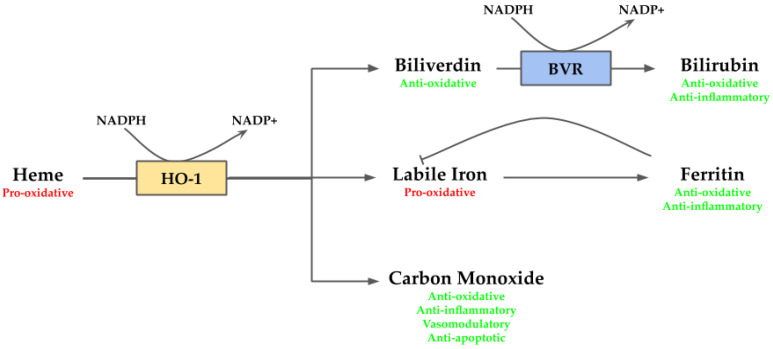
Overview of heme oxygenase (HO-1) system. Heme, a pro-oxidant molecule, is degraded by HO-1 into biliverdin, labile iron and carbon monoxide (CO). Labile iron can act as pro-oxidant through Fenton reaction, but it stimulates ferritin transcription, which stores it exerting anti-oxidative and anti-inflammatory activities. CO takes part in several downstream processes and eventually may act as anti-oxidative, anti-inflammatory, vasomodulatory and anti-apoptotic agent. Biliverdin is an anti-oxidative molecule, which is rapidly converted by biliverdin reductase (BVR) into bilirubin providing anti-oxidative and anti-inflammatory functions.

Heme is an essential molecule since it works as a prosthetic group for several hemoproteins, such as hemoglobin, cytochrome c oxidase, and peroxidases. However, in its free non-protein bound form, it is highly toxic. In this form, heme acts as a pro-oxidant molecule provoking oxidative damage via ROS generation [21]. In addition, the oxidative damage may trigger heme release from hemoproteins, which in turn may contribute to boost the oxidative state [21]. Along with its pro-oxidant properties, heme also exhibits pro-inflammatory behavior which contributes to cellular stress [42]. Given the adverse environment to the cells generated by heme, its removal is necessary to maintain homeostasis within the cell. This reaction is mediated by HO-1, releasing biliverdin/bilirubin, CO, and labile iron. These by-products also act as cytoprotective agents. The bile pigments biliverdin/bilirubin act as potent antioxidants [43]; CO exerts anti-apoptotic, anti-inflammatory and vasomodulatory activities [44]; and labile iron, which exerts anti-inflammatory function via ferritin [45], although also may produce deleterious effects since it is pro-oxidative [46] (Figure 2). 

### 3.1. Biliverdin/Bilirubin

The bile pigments have been considered as waste and toxic products derived from heme catabolism for so long, partly due to negative effects observed in hyperbilirubinemia conditions [47]. Intriguingly, bilirubin is a potent antioxidant, which has gained a relevant role for several pathologies in the recent few years [43]. Biliverdin and bilirubin may form a stable redox cycle which contributes to their role as antioxidant defense, since biliverdin is reduced to bilirubin, with higher antioxidant capacity, and then it could be re-oxidized to biliverdin again to close the cycle [48]. However, the existence of this redox cycle is still not clear and several studies claim that if it does, it has a limited role in antioxidant protection [49].

At low concentrations, bilirubin efficiently scavenges peroxyl radicals, and at serum levels, unconjugated bilirubin is able to prevent oxidative damage of LDL better than other known antioxidants such as vitamin E in vitro [50,51]. However, despite of bilirubin ROS scavenger capacity, its intracellular concentrations (about 20–50 nM) are too low to cope with the levels of ROS to which most cells are exposed, so its antioxidant protection has to be complement by other antioxidants that occurs at higher concentrations, such as glutathione [52]. The importance of bilirubin as antioxidant is that apart from acting as a direct ROS scavenger, its main physiological role at intracellular levels is to inhibit certain isoforms of NADPH oxidases [53]. These are membrane-bound enzyme complexes that catalyze the generation of superoxide free radical, mainly in phagosomes, but also in other several cell types [54]. Thus, bilirubin exerts its role as antioxidant by two different ways: Scavenging directly ROS along with other antioxidants, and inhibiting NADPH oxidases which are one of the major sources of ROS.

Bilirubin and biliverdin also have a role as endogenous immunomodulatory agents. Both bile pigments inhibit transendothelial leukocyte migration in vitro. Bilirubin at normal serums levels disrupts VCAM-1 signaling, blocking VCAM-1-dependent lymphocyte migration and ameliorating VCAM-1 inflammation by means of suppress derived ROS production [55].

Recent epidemiological studies highlight the potential of bilirubin to be employed as therapeutic target for stress-related diseases, since high levels of bilirubin have been associated with lower risk of stroke [56], diabetes [57], and non-alcoholic fatty liver disease [58].

### 3.2. Carbon Monoxide

For decades, CO has been considered as a poisonous molecule due to its strong affinity to hemoglobin, forming carboxyhemoglobin, which hampers oxygen delivery. The fact that it is endogenously produced in every mammalian cell, by means of HO, stands against the toxic effects and suggests a physiological function. Indeed, CO possesses several cytoprotective activities [44].

CO is a gas, so it can diffuse through all membranes freely allowing rapid responses within the cell as a signaling mediator or as regulator. From a chemical view, it is an inert gas which binds to transition metals present in proteins, but mainly heme-containing proteins [59]. As well as bilirubin, CO targets NADPH oxidase, a heme containing protein, inhibiting it and so, its oxidative-derived ROS [60]. In this context, CO exerts an antioxidant activity, but also an anti-apoptotic one in cerebral vascular endothelial cells by inhibiting apoptosis caused by TNF-α stimulation [61].

Another cytoprotective role of CO is mediated by its interaction with cytochrome c-oxidase, another heme-protein. CO at endogenous levels inhibits cytochrome c-oxidase, slowing down electron transport chain, which slightly increases mitochondrial ROS production. Although it may result counteractive, known the protective role of CO, this controlled ROS production act as signaling molecules, essential for CO mediated properties [62]. This controlled ROS increase may activate the p38 MAPK signal transduction pathway, which eventually leads to cytoprotection [62]. Indeed, CO triggers p38α (pro-apoptotic) degradation and favors p38β (anti-apoptotic) signaling pathway [63]. Beyond the anti-apoptotic properties mediated by MAPK pathway, its activation also leads to anti-inflammatory actions. CO derived activation of MAPK pathway results in the downregulation of pro-inflammatory cytokines, such as TNFα, IL-1β, and IL-6, along with the upregulation of the anti-inflammatory cytokine IL-10 [64]. MAPK signaling activation derived from CO also conduces to anti-proliferative actions mediated by downregulation of ERK, and an increase and decrease of p21 and cyclin D1, respectively [65]. 

Another target of CO is soluble guanylyl cyclase. Like NO, CO promotes the generation of cGMP which is linked with vasodilation, anti-thrombosis, and anti-proliferative activities [66,67]. 

CO also promotes angiogenesis through the stimulation of vascular endothelial growth factor (VEGF) production by increasing the transcription and stabilizing the hypoxia-inducible transcriptional factor-1 (HIF-1) [68]. 

The general antioxidant response within the cell is ensured by CO through stimulation of pentose phosphate pathway (PPP). Glutathione (GSH) is the major antioxidant, but its function depends on the recycling of its reduced form which is determined by the NADPH levels. At stress conditions NADPH levels decreases. CO promotes directly or indirectly the shift from glycolysis to PPP in order to provide amounts of NADPH required to limit oxidative stress [44]. 

### 3.3. Iron and Ferroptosis

The last product derived from HO-1 reaction is labile iron. Free iron, in its redox-active form, is a potent pro-oxidant, leading to ROS production through Fenton reaction which are deleterious to the cells [46]. In order to neutralize this increase of pro-oxidative free iron pool, the cell possesses two main mechanisms: By increasing intracellular iron storage in a non-toxic form within ferritin complex [69], and by an increase of ferroportin, which effluxes iron outside the cell [70]. Upon an increase of intracellular free iron and/or stress conditions, the transcription of ferritin is induced, as well as its post-transcriptional regulation is favored, reducing the pro-oxidant iron levels and so, providing cytoprotective properties [69,71]. The cytoprotective action from ferritin does not come just from sequestering free iron, but also it indirectly reduces TNFα-induced apoptosis along with NF-κB [72]. In addition, ferritin has iron-independent roles, since it has also been found in the nucleus where it may work protecting DNA from oxidative damage [73], and by downregulating JNK signaling involved in promoting apoptosis in response to damage [74]. 

Despite of the possible protective roles derived from HO-1 action, its over-activation may lead to an iron-dependent death, known as ferroptosis, as a result of its accumulation. Ferroptosis is a novel form of regulated cell death which is morphologically, genetically and mechanistically distinctive from apoptosis and necrosis [75]. It is the result of iron accumulation which contributes to lipid peroxidation and glutathione depletion [75]. The leading cause of ferroptosis can be multiple but stress conditions play a key part of them. Regarding this, HO-1 may be both protective or detrimental [76,77]. At low or moderate HO-1 activity, it provides a cytoprotective role since ROS are neutralized, and the iron generated can be taken into a non-pro-oxidative state. However, when HO-1 is highly activated, the NADPH levels decrease affecting to the antioxidant response; as well as the elevated iron levels which produced overwhelm ferritin neutralizing-effects over labile iron [76,78]. In addition, elevated ROS amounts also take a directly action over the ferritin overwhelm on the account of ferritin degradation by ROS-stimulated autophagy [79]. Independently of the mechanism, the inability to counteract the oxidative iron pool increases the amount of ROS and HO-1 activity, and the depletion of antioxidants, such as glutathione [75,76,78]. Together, the different mechanisms eventually lead to ferroptosis. 

## 4. The Central Role of HO-1 in Gastrointestinal Tract Diseases

The gastrointestinal tract is a complex organ system composed by several organs from the esophagus to the gut including stomach, pancreas and liver. The gastrointestinal tract itself is comprised by certain exclusive organs, beyond their main roles in food ingestion, digestion, and derived-waste expulsion; since they are continuously exposed to potential inflammatory stimuli such as food antigens, commensal microbiota, and drugs. Under physiological conditions, the exposition to these stimuli does not result in an unwarranted inflammation and stress condition because of the tight crosstalk among the microbiota, the immune system and diverse organs. In this context, HO-1 seems to be a key regulator of the gastrointestinal environment balance, since HO-1 activity is associated with several inflammatory and stress gastrointestinal injuries, such as diabetes [80], inflammatory bowel disease [81], and fatty liver diseases [82] that will be discussed in this section.

### 4.1. HO-1 and Esophagus

The esophagus is an organ of the upper parts of the digestive system, which is continuously exposed to external, but especially to internal oxidative agents from the stomach. An increment of those stimuli may lead to inflammation of the esophagus, and, ultimately, derived into disorders.

#### Esophagitis

Esophagitis is a disease defined by esophageal inflammation. Esophagitis can be developed due to many causes, but the most common is gastroesophageal reflux disease (GERD) [83]. There may be several causes underpinning GERD such as gastric acid secretion and abnormalities in esophageal defense mechanisms or in anti-reflux barrier. However, it is widely accepted that the common factor is exposure of esophageal tissue to ROS and acid, which eventually leads to an inflammatory state [83,84]. In this inflammatory context, some groups have found a protective role mediated by HO-1 and its by-product CO [85,86]. Kwon and colleagues found a decreased expression of Nrf2 and HO-1 in their esophagitis murine model compared with control rats; however, Magierowska and collages found that Nrf2 and HO-1 were upregulated in theirs, suggesting a self-defensive, but overwhelmed, mechanism to counteract the damaging effects [85,86]. In both models, the esophageal tissue showed an increment of ROS and pro-inflammatory responses mediated by an increase of NF-κB, MAPK-related proteins, and a cocktail of pro-inflammatory cytokines such as TNF-α, IL6, and IL1, which resulted in esophageal damage [85,86]. Upon pre-treatment with Rhe Rhizoma, Kwon and colleagues observed an upregulation of Nrf2/HO-1 pathway; however, Magierowska and collages did not see any increment in HO-1 using a pre-treatment with CORM-2, a CO releasing molecule [85,86]. These differences might be a direct consequence of different experimental animals and design. Despite of these differences, the final outcome was similar in both studies, reflecting an overall decrease of the inflammatory state due to a decreased expression of inflammation-related protein and ROS [85,86]. In the recent years, natural flavonoids have gained interest as a possible approach for GERD. Among them, isorhamnetin attenuates esophageal mucosal injury efficiently in a reflux esophagitis rat model with the possible mediated action of HO-1 to the protective action observed [87]. The administration of isorhamnetin provides antioxidant and anti-inflammatory actions through the suppression of NO generation, inhibition of p38 and NF-κB pathways, suppression of cytokines production and the infiltration of inflammatory cells, the reduction of oxidative stress markers, and HO-1 activity enhancement, which may be the orchestrator of the previous effects mentioned [87].

### 4.2. HO-1 and Stomach

The stomach releases gastric acid in order to initiate food digestion along with muscular contractions. The gastric acid, composed by digestive enzymes and hydrochloric acid, generates an acidic environment, which might derive in different diseases. 

#### 4.2.1. Peptic Ulcer Disease

Peptic ulcer disease (PUD) is the presence of painful sores or ulcers in the inner lining of the stomach, the lower esophagus or the first part of the small intestine, with a worldwide prevalence of 5–10% [88]. Traditionally it has been considered an acid-induced lesion as the result of a hypersecretory acidic environment along with stress and dietary factors. The main risk factors for PUD are the regularly use over an extended period of time of nonsteroidal anti-inflammatory drugs (NSAIDs) and *Helicobacter pylori* infections [88].

NSAIDs inhibit prostaglandins synthesis through COX-1 inhibition; however, that inhibition by its own, it is not enough to result in PUD. Nevertheless, it increases neutrophil adherence, which leads to reduced gastric blood flow and cytokine and ROS release, contributing to PUD [89]. One of the treatments for both, NSAIDs and *H. pylori* derived ulcers, is proton pump inhibitors (PPIs) because they inhibit acid secretion [88]. PPIs also seems to be therapeutic because they induce, independently of acid inhibition, HO-1 expression in gastric and endothelial cells. This in turn would improve gastric microcirculation, inhibit neutrophil adhesion and reduce inflammation and ROS through its by-products [90]. NSAIDs may also exerted their gastric epithelial cell damage by altering lipid raft organization and disrupting epithelial barrier, which ultimately lead to inflammation and oxidative stress [91]. In this context, a work tried to employ omega-3 fatty acids in order to counteract the adverse effects of NSAIDs by stabilizing the gastric cells membranes. It was observed omega-3 fatty acids were effective against indomethacin-induced gastric damage by inhibiting pro-inflammatory chemokines and cytokines, but also by increasing HO-1 levels; suggesting that omega-3 fatty acids based NSAIDs should be developed as a next generation of gastrointestinal-safe NSAIDs [91]. Interestingly, NSAIDs themselves also may induce HO-1 expression to protect gastric mucosa from NSAID-induced apoptosis, since inhibitor of HO-stimulated NSAID exacerbates ulcers [92]. In fact, several studies describe how the induction of HO-1 ameliorates peptic ulcers by means of the reduction of pro-inflammatory cytokines, cell adhesion, and stimulation of anti-oxidative state [89,93]. 

Furthermore, *H. pylori* infection of the stomach causes gastric mucosal inflammation and damage mediated by overproduction of ROS, neutrophil infiltration and IL-8 and NO production via NF-κB [94]. The use of diverse compounds with anti-inflammatory, anti-oxidative and antimicrobial properties improve patient outcomes [88]. Interestingly, resveratrol exerts beneficial effects suppressing IL-8, NO, and NF-κB, without no effect on *H. pylori* colonization, concurrently with an upregulation of Nrf2/HO-1 pathway, evidencing once more the important protective role of HO-1 in the gastrointestinal tract [94]. A study carried out by Lee et al., found that *L. plantarum* isolated from kimchi, a traditional Korean fermented side-dish, exerts anti-inflammatory actions against *H. pylori* [95]. A more recent work by the same group points out that the dietary intake of kimchi significantly increases the expression of HO-1, and its long term consumption ameliorates chronic *H. pylori* associated gastritis and prevents *H. pylori*-induced gastric tumorigenesis [96]. Among the ingredients of kimchi stand out pepper, black cumin, rosemary, bay leaves, or garlic, inter alia, which are rich in carotenoids and flavonoids associated with anti-oxidative and anti-inflammatory responses [96]. Concerning to garlic, organic garlic compounds like S-allyl cysteine induces HO-1 expression and may be used to alleviate NSAIDs-induced ulcers [93], and so may contribute to the amelioration of *H. pylori* associated gastritis observed by Jeong et al. [96].

#### 4.2.2. Gastroparesis

Gastric motility is an important event for two main reasons, facilitating food crushing and for gastric emptying towards the small intestine. An abnormal motility pattern may be the consequence of several illnesses. Gastroparesis is a disorder caused by a weak muscular contraction of the stomach which results in a delayed gastric emptying of solid food. Gastroparesis may be idiopathic, associated with diabetes or neurological disorders, and may occur after infections or medical interventions [97]. The mechanism and pathophysiology of gastroparesis are still not well understood with differences depending on its cause. For this reason, in this section we will focus on the role of interstitial cells of Cajal (ICC), since they act a pacemaker by transducing excitatory or inhibitory neural signals to produce electrical rhythm in the smooth muscle cells of stomach [97]. Loss of ICC has been observed in idiopathic and diabetic gastroparesis [98]. 

In diabetic murine models, there is an increase of oxidative stress following diabetes development, which leads to HO-1 upregulation. If the latter is lost by some reason, loss of ICCs occurs and mice develop delayed gastric emptying, but if it is induced back, the gastroparesis phenotype is reversed upon decrease in ROS, an increase of neural NO and ICC [99]. The damage to ICCs is due to dominance of macrophages in the activated M1 form, which are led into the anti-inflammatory M2 form upon HO-1 induction by hemin [100]. The reversal phenotype observed with HO-1 induction may be mediated in last term by CO [101]. However, when HO-1 induction by hemin was brought to humans, it failed to sustain HO-1 levels beyond a week, with weekly hemin administration, and did not improve gastroparesis compared to placebo [102]. 

### 4.3. HO-1 and Pancreas

The pancreas is an organ with endocrine and exocrine function. As an exocrine organ, it secretes pancreatic juice into the duodenum to continue the digestive process. The endocrine function is exerted by the islet of Langerhans, which secrete glucagon and insulin affecting to glucose metabolism. Pancreatic cells, specially β-cells, are extremely sensitive to oxidative stress, so under certain circumstances it may lead to oxidative damage and inflammation [103]. 

#### 4.3.1. Pancreatitis

Pancreatitis is an inflammatory disease divided into two clinical states: Acute and chronic pancreatitis. Acute pancreatitis (AP) is a quite common disorder in US, and its incidence is increasing in Europe [104]. AP manifestations range from asymptomatic or mild transient self-limited inflammatory reaction to severe organ dysfunction with high mortality associated with necrotizing AP [104]. On the other hand, chronic pancreatitis (CP) is characterized by a chronic progressive pancreatic inflammation, which may be driven by recurrent AP, associated with several complications, and as a potential risk for pancreatic cancer [105].

Regarding HO-1, it is upregulated in pancreatitis murine models [106], and in peripheral blood mononuclear cells (PBMC) from AP patients [107]. Indeed, patients with necrotizing AP, the most severe form of AP, more frequently are carriers of long GT repeats genotype compared with those who has mild AP, or with healthy patients, associated with lower HO-1 expression [108]. 

The inflammatory state in pancreatitis seems to be triggered by a premature activation of pancreatic proteases which leads to pancreas autodigestion. This eventually leads to generation of ROS, necrosis and inflammation [109]. In this scenario, NF-κB mediates an inflammatory response interceded by pro-inflammatory mediators such as TNF-α, IL-6, IL-8, monocyte chemoattractant protein-1, macrophage inflammatory-1, and adhesion molecules, which lead to immune cells recruitment. The release of pro-inflammatory mediators promotes an anti-inflammatory response, and if it is adequate, the patient recovers, but if not, the pancreatitis gets exacerbated [109]. The HO-1 upregulation noted in pancreatitis seems to be one of the anti-inflammatory responses, since HO-1 upregulation in monocytes during the course of AP was decreased as the patient recovers [107]. The HO-1 induction by hemin increases the amount of macrophages overexpressing HO-1 recruited into de pancreas, protecting it from the damage. However, it only accounts as prophylactic for high-risk AP patients since it failed to protect when it was administered once AP was already developed [110]. Despite that, a recent study shows how the administration of hemin in early AP increases HO-1 expression, reducing pancreatic damage and TNF-α levels, and increasing the release of anti-inflammatory IL-10 by macrophages, stellate cells and T helper type 2 cells [111]. Administration of bilirubin or CO protects against pancreatitis by inhibiting NF-κB inflammatory responses and increasing the Nrf2/HO-1 pathway [112], or by impairing the recruitment of pro-inflammatory immune cells [113], respectively. 

#### 4.3.2. Diabetes

Pancreatic β-cells are extremely sensitive to oxidative damage due to their elevated production of ROS and the relatively low levels of antioxidants defenses, which may explain the contribution of oxidative stress to the development of diabetes [103]. Diabetes is a metabolic disease characterized by chronic hyperglycemia as a result of defects in insulin production or insulin sensibility, which will account for more than 592 million cases by 2035 worldwide [114]. It is classified into type I diabetes mellitus (T1DM), which is considered as an autoimmune disease caused by loss of pancreatic β-cells, and type II diabetes mellitus (T2DM), caused by insulin resistance, although it eventually leads to impairment in insulin production and β-cell death. In both cases, inflammatory events are the major contributors to the damage of the pancreatic islets [114]. Patients with T1DM show infiltration of T and B cells, macrophages, dendritic cells, NK cells, and islet-reactive T-cells in the pancreas [115]. Damaged β-cells release auto-antigens, which are presented by the antigen-presenting cells (APC) to T helper cells (Th), which promote inflammation. These together produce T-cells and macrophages activation, leading to β-cell death by effector T-cells, as well as β-cell apoptosis mediated by the release of ROS and pro-inflammatory cytokines, such as IL- β, INF-γ and TNF-α, which stimulates additional JNF and NF-κB inflammatory pathways [115]. On the other hand, T2DM also underlies inflammation, but it starts by different mediators. In T2DM, there is insulin resistance, which among others effects, leads to lipid metabolism impairment [116]. This lipid metabolism alteration together with hyperglycemia prompts β-cell-failure by means of reduced associated insulin genes expression and overall inflammation [115,116]. Additionally, fatty acids can promote insulin resistance by phosphorylation of insulin receptor substrate by inflammatory mediators such as TNF-α., MCP-1, IL-6, IL-1β, and PAI-1 [115]. In T2DM, there is a chronic systemic inflammation that impairs insulin secretion and sensitivity, which eventually may lead to loss of β-cells, exacerbating even more, the disease progression [115,116,117].

In this context, protecting β-cell from the inflammatory and oxidative environment, and so from their death, may be a way to treat or reduce diabetes. Indeed, HO-1 due to its anti-apoptotic, anti-oxidative, and anti-inflammatory properties seems to play an important role for preventing diabetes. 

As well as in other inflammatory diseases, individuals carrying longer GT repeats at *HMOX1* promoter are more likely to develop T2DM [118]. Additionally, higher HO-1 by-product bilirubin levels are associated with lower risk for diabetes, supporting the HO-1 protective action [119]. The induction of HO-1 exerts beneficial effects by two different mechanisms. One of them is through the potentiation of insulin-sensitization and glucose metabolism agents such as GLUT4, adiponectin, AMPK, cAMP, and cGMP, as well as an increase in antioxidants levels [120]. In the same study, pro-inflammatory mediators such as NF-κB were reduced, alleviating the suppression of those mediators on insulin biosynthesis, release and response to [120]. A second beneficial effect of HO-1 induction occurs through the recruitment of anti-inflammatory M2 macrophages which forms aggregates consisting of M2 macrophages, mesenchymal cells and fibrocytes. These aggregates seem to promote a pro-survival environment enhancing an anti-inflammatory state and pancreatic islet regenerative processes [80]. A recent study highlights that NOD mice, a common murine model for diabetes, exhibit lower amount of dendritic cell (DC) expressing HO-1. This type of APC is responsible of autoreactive T-cell activation in T1DM [121]. Interestingly, the authors observed how the restoration of HO-1 expression reduced the incidence of T1DM [121]. An alternative option to HO-1 induction may be the use of its by-product CO. A recent work describes the development of a biomimetic CO nanogenerator loaded with Manganese Carbonyl (MnCO) and camouflaged with macrophage membrane for diabetes treatment [122]. In that way, the CO nanogenerator evades the immune system and is accumulated to the pancreas by the active targeting of macrophage membrane to inflammatory sites. Once there, ROS within the pancreas trigger the continuous CO release, which reduces apoptosis of β-cell and inflammation markers, and improves glucose and insulin levels [122]. The results were better when it was used as prophylactic, but they were also significant after the disease was established [122].

Together, these studies demonstrate the relevant role of HO-1 in diabetes development, and the potential of HO-1 induction for both preventative and management approach for diabetes.

### 4.4. HO-1 and Liver

The liver is an organ involved in the detoxification process, as well as protein synthesis and secretion of bile in order to help in fat digestion. Several hepatic disorders may lead to liver deterioration with an important impact on patient’s lifestyle. In fact, the loss of liver function cannot be compensated, being liver transplantation the only effective treatment once liver completely failure occurs.

#### 4.4.1. Non-Alcoholic Fatty Liver Disease

Non-alcoholic fatty liver disease (NAFLD) comprises a wide spectrum of conditions in which there is an accumulation of excess fat in the liver of individuals with low or no consumption of alcohol, with a prevalence up to 25% worldwide [123]. NAFLD ranges from the less severe and most common form called fatty liver characterized by accumulation of fat in hepatic cells, to the most severe form called non-alcoholic steatohepatitis (NASH) which includes inflammation and fibrosis, leading to cirrhosis [123].

Although the intrinsic mechanism is complex, it is widely accepted that the main driver of NAFLD is oxidative stress. The oxidative stress is the result of increased levels of free fatty acids and impairment of mitochondrial respiratory chain within hepatocytes, and the subsequent lipid peroxidation, ROS production, antioxidant enzymes depletion and upregulation of pro-inflammatory cytokines [124]. Upon continuous damage, hepatic stellate cells (HSCs) are activated, and, in this sense, the liver parenchyma responds by producing an excess of extracellular matrix, and latter causing fibrosis [124]. In fact, an oxidative state is prominent in NAFLD and NASH [125]. The expression of HO-1 is increased in NAFLD, and is associated with the severity of the disease [82]. In the latter study, the increment of HO-1 following the severity of the disease may suggest a pathophysiological role of HO-1 in NAFLD, however, as in other inflammatory diseases, it is a protective defense [126]. In vitro, induction of HO-1 by hemin caused a decrease of alanine aminotransferase and triglycerides release, markers of abnormal liver function, and oxidative stress; but the use of HO-1 inhibitors impaired the previous protective effect [126]. The experiments in vivo also reflected the protective effect by means of upregulation of antioxidant defenses such as Crya chaperone, glutathione, superoxide dismutase, and Fancc, which decrease ROS levels, protect against apoptosis and necrosis, and suppress inflammatory cytokines [126]. A recent study pointed out that HO-1 expression may protect hepatocytes by suppressing ROS-dependent endoplasmic reticulum stress and related apoptosis [127]. On the other hand, HO-1 has an important impact over HSCs, which are the major contributors to hepatic fibrosis [124]. In response to continuous damage, HSCs overexpress α-smooth muscle actin (α-SMA), undergoing proliferation and activation, and promoting collagen secretion, which eventually result in fibrosis. HO-1 induction decreases α-SMA expression, collagen synthesis, and reverses liver fibrosis by inhibiting NF-κB and promoting PPARγ pathways [128]. In addition, HO-1 upregulation decreases Bcl-2 and increases Caspase-3 levels in HSCs, promoting their apoptosis, likely by inhibiting NF-κB activity [129].

#### 4.4.2. Hepatic Ischemia–Reperfusion Injury

Liver ischemia–reperfusion injury (IRI) is the result of different medical states ranging from trauma and sepsis to liver transplantation. Indeed, orthotopic liver transplantation (OLT), the standard life-saving strategy for patients with liver failure, is not just a cause for IRI, but also the major risk for liver graft rejection [130]. The IRI observed in OLT is the result of two different, but coupled processes. In first place, “cold” IRI, being characterized by damage to hepatic sinusoidal endothelial cells and microcirculation disturbance during the preservation stage. On the other hand, “warm” IRI occurs as the result of hepatocellular damage during the transplantation of the cold-preserved liver into the organ recipient [130]. Initially, the blood flow ceasing and the subsequent lack of oxygen supply generate the ischemic injury as a result of metabolic stress due to glycogen and ATP depletion, leading to parenchymal cell death. Hence, damage-associated molecular patterns (DAMPs) are released initiating a sterile inflammation. During reperfusion, the cells may recover from the ischemic insult, but they generate ROS which aggravates the inflammatory immune response [130]. Macrophages, Kupffer cells, neutrophils and T-cells involved release pro-inflammatory cytokines, ROS and vascular cell adhesion molecules which exacerbate the immune response and eventually contribute to hepatocellular damage. In this sense, HO-1 has been reported to have a protective role in hepatic IRI [131]. In humans, livers with low HO-1 expression before transplantation correlate with less posttransplantation injury, suggesting that the induction of HO-1 during transplantation is more protective than elevated HO-1 expression in pre-transplant liver [132]. In accordance with the latter, a more recent work found that liver macrophages are the major HO-1 producers, and that high expression in human OLT at reperfusion phase are associated with better survival and hepatocellular function [133]. HO-1 seems to exert cytoprotective action through different mechanisms based on reduce inflammatory response and oxidative stress. During IRI, HMGB1 is released from damaged hepatocytes and sinusoidal endothelial cells initiating TLR4 and IFN-γ signaling, which promote M1 phenotype macrophages. This polarization stimulates a pro-inflammatory environment as a result of TNFα, IL-6, IL-12, or ROS release, which induce a Th1-type response [130]. In this regard, low levels of HO-1 are associated with an increase of M1 macrophages markers, as well as, HO-1 expression polarizates M2 macrophages with anti-inflammatory responses in liver IRI [134]. The overexpression of HO-1 downregulates the TLR4 pathway reducing the immune response and exerting cytoprotective and anti-inflammatory functions [135]. This role is supported by a study in which the deficiency of ATF3, a stress-induced transcription factor, depressed HO-1 signaling conducing to a TLR4-driven inflammatory response and liver injury increase [136]. An additional cytoprotective role in IRI may be exerted by HO-1 through the expansion of regulatory T cells (Treg) and reduction of CD4 and CD8 T cell infiltration [137]. Liver macrophages are the main source of HO-1, suggesting this cell type could be used to reduce the risk for IRI after transplantation [133]. Indeed, native macrophages transfected ex vivo with HO-1 and applied at time of transplantation diminish liver injury, emerging as a possible strategy for hepatic IRI [138]. Recently, it has been described that HO-1 enhances autophagy in hepatic IRI, and it seems to occur in a SIRT-1 dependent manner [139]. This point out the relevance of HO-1 cytoprotection and suggest SIRT-1 as a new possible target for hepatic IRI.

### 4.5. HO-1 and Small and Large Intestine

The small and large intestine encompass the last part of the gastrointestinal system, where highest chemical digestion takes places, and where most nutrients, minerals and water are absorbed. The large intestine works as well as storage of the remaining waste material before its removal. One of the most striking fact about the gut is that it is colonized by a complex and dynamic population of microorganisms, comprising the so-called gut microbiota. The immune system tolerates the gut microbiota maintaining a continue cross-talk between them, so an imbalance in these microbiota-immunity interactions under certain environmental context may lead to oxidative stress and the development of inflammatory diseases [140].

#### 4.5.1. Inflammatory Bowel Disease

Inflammatory bowel disease (IBD) is a group of chronic inflammatory conditions of the colon and small intestine. They are classified into Chron’s disease (CD) and ulcerative colitis (UC), which have a high prevalence in Western countries and are increasing in developing countries, associated with substantial patient burden and cost to health care systems [141]. CD inflammation can occur in any section of the gastrointestinal tract, meanwhile UC primary does in the colon and rectum. Although the main cause of IBD has not yet been elucidated, the inflammation of the gastrointestinal mucosa underlies the recruitment of neutrophils and macrophages that produces cytokines, proteolytic enzymes and ROS which lead to inflammation and ulceration [142]. Under physiological conditions, the intestinal mucosa exists in a balanced equilibrium with the surrounding environment. The intestinal mucosa is formed by intestinal epithelial cells (IECs) and complemented by dendritic cells, macrophages, innate lymphoid cells and neutrophils. Briefly, in the healthy gut, the macrophages population is primary composed by the alternative activated macrophages (M2) population with attenuated proliferation and hyporesponsiveness state, and by a polarization towards Treg instead of T helper cells, that promote a tight controlled anti-inflammatory state through production of IL-10 [141]. However, in IBD, this cross talking is impaired, likely by microbial dysbiosis along with other genetics and environmental factors [142]. This impairment results in loss of the gastrointestinal barrier integrity and an imbalance between pro- and anti-inflammatory responses. IBD patients display an activated mucosal immune system with a high recruitment of neutrophils, macrophages, and overexpression of cytokines and chemokines with recruit the inflammatory cells [141]. There is a chronic inflammation in both CD and UC but is slightly different. In CD, it is characterized by an impaired Th1 and Th17 responses which are triggered by pro-inflammatory cytokines such as TNF-α, IL-12, IL-17, IL-18, and IFN-γ [141,142]. Meanwhile, in UC the inflammatory state is mediated by a Th2 response and production of IL-4, IL-5, and IL-13. Independently of the type of immune response, the overall response is mediated by an inappropriate mucosal immune response against the gut microbiota by means of polarization from Treg towards Th cells promoting an inflammatory state [141,142].

HO-1 seems to play an important role in IBD development since it is upregulated in the affected colonic mucosa of UC patients compared to their normal mucosa [81]. This same evidence was found in other study, even when compared to healthy non-inflamed controls [143]. In this study, they observed the same effects on murine dextran sulfate sodium (DSS)-colitis model. Interestingly, they found how the induction of HO-1 before the onset of inflammation ameliorates colitis, although the induction has no effects once inflammation is already established [143]. In this context, HO-1 may provide protection against UC development, confirmed by a higher susceptibility to UC in mice lacking the HO-1 transcriptional factor Nrf2 [144]. On the other hand, inhibition of HO-1 repressor Bach1 in 2,4,6-trinitrobenzene sulfonic acid (TNBS) induced colitis model, resulted in HO-1 expression on macrophages. This HO-1 upregulation polarized the macrophages subpopulation towards M2 macrophages, inhibiting colitis. Interestingly, when these M2 macrophages were transferred into wild type TNBS-induced colitis mice, colitis was inhibited [145]. These results suggest that HO-1 induction or HO-1 by-products administration may be used as treatment for IBD. Indeed, bilirubin administration ameliorates UC by inhibiting NF-κB pro-inflammatory pathways and stabilizing intestinal flora [146]. Curiously, some of the current treatments for IBM, such as 5-aminosalicylic acid, exert their therapeutic actions, at least in part, through the upregulation of HO-1 [147]. However, since some IBD do not respond or they do partially, in the last few years has been a special interest in the discovery of compounds that are able to upregulated HO-1, and so provide anti-inflammatory actions. In this sense, several studies have claimed how artemisinin derivatives ameliorate IBM via decreasing pro-inflammatory cytokines, inhibiting NF-κB, promoting Th cell apoptosis and increasing Treg cells, through HO-1 upregulation, and so promoting an anti-inflammatory state [148,149]. Similar effects over the regulation of Th/Treg balance, mediated by HO-1 induction, have been observed after the administration of others plant extracts from oriental medicine, such as *Atractylodes macrocephala* and *Taraxacum herba* [150], and *Angelica gigas* [151,152]. Oligonol, a polyphenol derived from the Chinese lychee fruit, ameliorates colitis in DSS-induced colitis model, but, interestingly, Oligonol prevents IBD relapse better than sulfasalazine [153]. The mechanism by this protection takes places seems to be mediated by Nrf2 enhancement resulting in Nf-κB pathway inhibition and an upregulation of anti-oxidant enzymes such as HO-1 [153]. The enhancement of Nrf2 pathway, and the subsequent increase of HO-1 levels and the inhibition of Nf-κB activation are effects observed in several pre-clinical models of IBD after the administration of others herbal-derivate compounds such as Genipin [154], Toosendanin [155], or *A.japonica* extracted ethanol [156]. Overall, these natural compound derivative administration results in a significant increment of HO-1 levels, and in the decrease of pro-inflammatory mediators release, reduced ROS production, increment of anti-inflammatory cytokines and better intestinal barrier function along with less colonic tissue injury [154,155,156]. There are others compounds, distinct from those derivative from plant extracts that also exert anti-colitic effects by means of Nrf2/HO-1 pathway, such as FA-97, a synthetic caffeic acid phenethyl ester derivative, and Galangin, both found on honeybee propolis [157,158]. However, all the previous data are from pre-clinical models, so clinical trials are required to confirm the use of these natural compound derivatives in human patients with IBD.

#### 4.5.2. Post-Operative Ileus

Intestinal motility is an important process to facilitate digestion and absorption of nutrients, and the elimination of feces. Dysfunction of this process is associated with different afflictions. Post-operative ileus (POI) is a condition characterized by a decrease or arrest in intestinal motility following a gastrointestinal surgery. When POI is prolonged in time, it is a cause of patient morbidity, lengthening hospital stay and increasing hospital costs. For instance, just only for the US economy it accounts for $1.46 billion [159]. The main cause of POI seems to be an inflammatory cascade within the intestinal muscularis upon the manipulation of the bowel. As a result of the inflammatory cascade, pro-inflammatory cytokines and chemokines are released, which increase the recruitment of leukocytes, which in turn produce NO and prostaglandins with inhibitory outcomes on the neuromuscular smooth muscle contraction [159]. The application of low exogenous CO levels attenuate POI in murine models improving the contractile function [160]. The pretreatment with CO increases the expression of anti-inflammatory pathways mediated by HO-1 and IL-10, the latter inhibits the production of pro-inflammatory TNFα, IL-6, and IL-1; and dampens the pro-inflammatory IL-6 pathways, as well as decreases NO production and IL-1β, which inhibit smooth muscle contraction [160]. Another study carried out by De backer and colleagues showed similar effects using water-soluble-CO releasing molecules, but in this case the protective effects described are mediated in part by a p38-dependent induction of HO-1 and a decrease of ERK1/2 activation [161]. A more recent study used hemin pre-treatment as POI therapy, improving digestive motility through an increase of HO-1, and a reduction of leukocyte infiltration and IL-6 [162].

## 5. HO-1 in Gastrointestinal Cancers

Despite of the cytoprotective actions described in the previous sections, HO-1 and its by-products may be cytotoxic in a tumorigenic environment. Indeed, HO-1 is overexpressed in several gastrointestinal cancers including colon [163], gastric [164], and pancreatic [165] cancer. Moreover, normally, high HO-1 expression is associated with poor prognosis, lower survival rate and, especially, less responsiveness to treatments [163,164,165]. However, there is some controversy in this sense, due to differences found among studies. For instance, in colorectal cancer, some studies highlight a better long-term survival and less metastases when HO-1 is expressed, and lower HO-1 expression at high grade stages [166]; meanwhile other studies observed that the high HO-1 expression in colorectal cancer cells is linked to tumor progression and metastasis, by inhibiting apoptosis and promoting angiogenesis and immunological scape [167]. Another study supports the protective role of HO-1 in colorectal cancer, describing high HO-1 expression in advanced stages and apoptotic HO-1 activities in collaboration with p53 [168]. However, when p53 was lacked, no apoptotic function was seen [168]. This may in part, at least in colorectal cancer, explaining differences among studies since p53 is mutated in over 50% of human cancer, suggesting that HO-1 effect on cancer may be dependent on p53 status.

**Figure 3:**
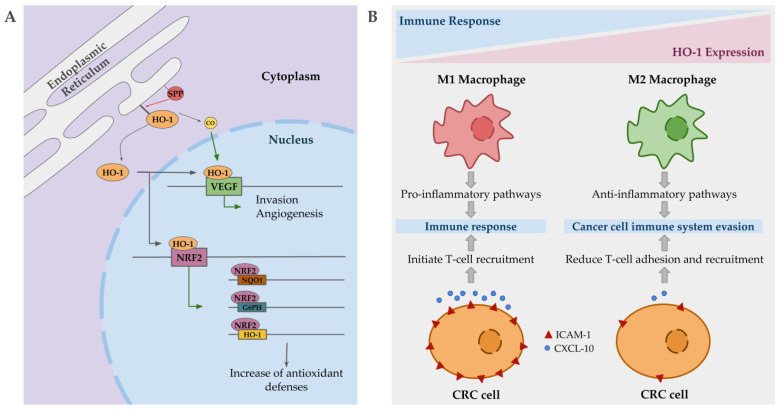
Mechanisms by which heme oxygenase 1 (HO-1) may contribute to cancer progression. (**A**) Signal peptidases (SPPs) mediate HO-1 compartmentation to the nucleus, where it regulates the expression of vascular endothelial growth factor (VEGF), promoting angiogenesis and invasion. CO, in a tumorigenic environment, may also promote the same previous effect. HO-1 also regulates nuclear erythroid 2-related factor (Nrf2) expression, which in turn regulates the expression of antioxidant enzymes such as HO-1, G6PH, and NQO1, enhancing the antioxidant capacities of the tumor cells. (**B**) HO-1 expressing macrophages are polarized into M2 state, promoting anti-inflammatory pathways and facilitating cancer cell to evade the immune system. In colorectal cancer (CRC) cells, the overexpression of HO-1 results in low levels of ICAM-1 and CXCL-10, reducing T-cell adhesion and recruitment.

Intriguingly, in cancer, nuclear localization of HO-1 has been described, being associated with poorer prognosis, cancer proliferation and invasion. This compartmentation of HO-1 is mediated by signal peptide peptidases (SPPs), which are also overexpressed in several cancer types [169]. Nuclear HO-1 seems to act as a transcriptional factor promoting the expression of VEGF in some cancer lines, promoting in turn angiogenesis and invasion [170]. Additionally, VEFG expression is also promoted by CO, in order to facilitate wound healing, but in a tumorigenic environment it may contribute to the harmful effects associated with HO-1 upregulation by enhancing angiogenesis, proliferation and survival (Figure 3A) [171]. Although, some contradictory reports are found in reference to the role of CO, since several studies show antiangiogenic and anti-proliferative effects in pancreatic and colorectal cancers when it is applied exogenously [172,173]. Another gene positively regulated by nuclear HO-1 is Nrf2 [174]. This upregulation of Nrf2 results in a significant induction of NQO1, G6PH and HO-1 expression, which is translated into changes in metabolic routes promoting PPP, and an overall increase in anti-oxidative responses (Figure 3A) [174]. Indeed, Nrf2 is overexpressed in pancreatic and hepatic cancer [175,176], and mutations in its inhibitor, keap1, also have been described in colorectal cancer [177]. This major antioxidative capacity may be the responsible of resistance to chemotherapy based on induced stress oxidative damage to cancer cells.

Apart from the expression on tumor cells, elevated HO-1 expression has also been described in tumor-associated macrophages with an important impact on disease progression. Immune cells may infiltrate to tumor site and influence cancer progression. Macrophages, depending on their polarization state, may be able to kill tumor cells and initiate an immune response (M1 macrophages), or support invasion and metastasis (M2 macrophages) [178]. HO-1 positive macrophages are polarized into a M2 state, promoting anti-inflammatory pathways, allowing cancer cells to evade the immune system [179]. In colorectal cancer, HO-1-positive macrophages are more frequent in advanced tumors and are correlated with worse prognosis [180]. In addition, HO-1 expressing colorectal cancer cells stimulate immune evasion by reducing the expression of ICAM-1 and CXCL10, which in turn reduce effector T cell adhesion and recruitment (Figure 3B) [167]. Additionally, CO promotes the development of tolerogenic DC and inhibits effector T cells, at the same time that HO-1 favors Treg induction and inhibits T cell response, facilitating tumor cells to evade the immune system [181].

Due to the distinctive roles that HO-1 play in cancer progression, targeting it is one of the options propose as cancer treatment. Inhibition of HO-1 has been used for pancreatic cancer, enhancing the response to chemotherapy and reducing cell proliferation [182]. Similar effects have been observed in colorectal cancer and hepatocarcinoma upon the use of HO-1 inhibitors [183,184]. Additionally, the use of HO-1 inhibitors also may be used to inhibit metastasis, as seen in gastric cancer by inhibiting HO-1 angiogenic activity [185]. Controversially, some studies point out the induction of HO-1 stimulate pro-apoptotic effects through CO production and endoplasmic reticular stress [186]. Specially, the use of several natural compounds such as curcumin in combination with nobiletin [187], non-extractable polyphenols from cranberries [188], and Ginnalin A [189] have been proved to exert anti-tumor properties in colorectal cancer cell lines, with a significant induction of HO-1 expression. However, the antitumor properties of those natural compounds may not be a direct effect of HO-1 upregulation, but dependent on other pathways. In fact, photodynamic therapy as treatment for esophageal cancer induces apoptosis and autophagy through the generation of ROS, besides inducing HO-1, likely as a self-protection mechanism which is not enough to counteract the cytotoxicity generated by this therapy [190]. This kind of “self-protection” may be responsible of the increment of HO-1 instead of a protective effect arising from the antitumor compounds. Another interesting approach for cancer therapy is to induce HO-1 expression in order to promote ferroptosis in cancer cells due to its higher HO-1 expression [76,78]. Interestingly, ferroptosis seems to be regulated in part by p53 [191]. This fact along with the controversial results about the beneficial or detrimental effect of HO-1 could be attributed to the p53 status [168], opening the door to explain them in base of the sensibility of the tumor cell to ferroptosis. Indeed, further research in that sense need to be done, and it would be interesting to put HO-1 expression in cancer cells in a ferroptotic context.

## 6. Clinical Trials Involving HO-1

Despite the wide implication of HO-1 in the development, progression and on the effect of different treatments in the gastrointestinal diseases described above, the number of clinical trials performed in which this enzyme is used as a target is small. In this section, we aimed to describe such as clinical trials found in ClinicalTrials.gov, EU Clinical Trials Register (EU CTR), and the World Health Organization International Clinical Trials Registry Platform (WHO ICTRP) databases.

Studies carried out in healthy volunteers have focused on the effect of dietary supplements on the expression of HO-1 (Table 1), to finally demonstrate the possibility of their use as an alternative in the treatment of diseases in which dysregulation of HO-1 expression has been implicated in their development [192].

The pilot study NCT00895167 investigated the inducibility of HO-1 by orally administered curcumin in healthy male subjects and its correlation with the GT length polymorphism in the promoter region of the *HMOX1* gene, which regulates its expression [196]. Authors concluded that curcumin administration does not induce HO-1 on mRNA or protein level in PBMCs. In addition, there was no correlation between any of the parameters and GT length polymorphism [192]. Other studies used resveratrol (NCT01768507) or different plant concentrates not specified (NCT02213523), although none of them showed the results of the trials. Hemin and heme arginate are potent inductors of HO-1 [197,198] used mainly for the treatment of porphyria [199,200]. Clinical trials in healthy humans (NCT00882804, and NCT00682370) showed that both drugs increased the expression of HO-1 in plasma and peripheral blood monocytes, respectively [193,194]. Further, heme arginate exerts its effects in humans irrespective of the GT(n) genotype [194]. However, while aspirin, simvastatin and α lipoic acid activate HO-1 in vitro models, at therapeutic doses, these drugs do not upregulate HO-1 activity in healthy humans [195]. The histamine H2 receptor antagonist nizatidine and the ACE inhibitor Lisinopril were also evaluated in healthy subjects, although no results were posted (NCT02232308).

At present, few studies are analyzing or have analyzed HO-1 expression as a predictor of disease outcome (Table 2). The trial NCT00842205 concluded that microsatellite variations in the *HMOX1* gene are not associated with liver injury in chronic hepatitis C virus (HCV) after treatment with rivabirin and/or pegylated interferon [201]. The NCT01112033 case-control study also showed that HMOX1/2 does not seem to have any predictive potential in relation to therapeutic response in HCV patients [202]. In relation to liver diseases, the role of HO-1 induction in hepatic regeneration after partial hepatectomy (PH) is been evaluated (NCT04195438). 

In colorectal cancer, HO-1 activity has been evaluated as a part of the antioxidant capacity of patients to determine whether a defined exercise program can improve recovery and reduce complications after surgery (NCT02264496). However, short surgical wait times and patient engagement represented major obstacles to implementing exercise rehabilitation programs in colorectal cancer patients and to obtain applicable results [203]. In ulcerative colitis, an active although not-recruiting study is also using HO-1 expression as a marker of the antioxidant capacity of patients after hyperbaric oxygen treatment (NCT03494764).

Some strategies include the use of CO as new treatment (Table 3). At the NCT01050712 trial, researchers hypothesized that inhaling CO before and after colon surgery would shorten the length of normal post-operative ileus (POI), a temporary paralysis of the intestines that could appears after colon surgery, and decrease the occurrence of POI complications with minimal side effects. However, no results were published in relation to this study.

A pilot trial related to chronic pancreatitis (NCT02567240) showed for the first time that harvesting islets in carbon monoxide-saturated solutions is safe and can enhance islet survival and insulin independence after total pancreatectomy and islet autotransplantation [205].

Two clinical trials used hemin treatment to increase the expression of HO-1 and thus taking advantage of its protective effect (Table 3). The NCT01206582 trial is a pilot study designed to learn if hemin can increase the production of HO-1 and improve gastric (stomach) emptying and symptoms in diabetic patients with slow gastric emptying (gastroparesis). However, the treatment regimen used for hemin failed to sustain increased HO-1 levels beyond a week and did not improve gastric emptying or symptoms [102]. The NCT01855841 study is based on the fact that the activation of HO-1 by intraperitoneal administration of hemin leads to the prevention and treatment of acute pancreatitis in mice models [204]. One complication of ERCP (endoscopic retrograde cholangiopancreatography) is acute pancreatitis, which happens in 5 to 25% of cases. Thus, the aims of this study are to study the activation of HO-1 by hemin in humans and its protective effect in post-ERCP acute pancreatitis incidence and to use the human situation of post-ERCP acute pancreatitis as early pancreatitis model to study the administration of hemin as treatment of acute pancreatitis in general. This trial is still recruiting patients and, at the moment, it has no published results.

## 7. Conclusions

Despite the extensive research being carried out regarding the potent anti-inflammatory and antioxidative properties of the HO-1 system exerts on several disorders of the gastrointestinal tract, the transfer of current knowledge to clinical practice is being slow. There can be several causes for this. First, the number of clinical trials involving HO-1 itself or any of the by-products of heme breakdown is low. On the other hand, it is necessary to have more research focused in the development of new drugs able to regulate HO-1 expression and/or function. At present, new CO-releasing drugs are being developed which are proving to be promising tools for further investigation [206]. Finally, diseases such as cancer showed two distinct an opposite effects of HO-1 that makes necessary deeper basic research before making the transfer to the daily clinic successfully.

## Figures and Tables

**Table 1:** Clinical trials carried out in healthy volunteers.

ID ^1^	Phase	Intervention Model	Status	Interventions	Ref.
NCT00895167	1	Single Group Assignment	Completed	Dietary Supplement: Curcumin	[192]
NCT01768507	N/A ^2^	Parallel Assignment	Completed	Dietary Supplement: Resveratrol	-
NCT02213523	N/A ^2^	Parallel Assignment	Completed	Dietary Supplement: Plant concentrate A Dietary Supplement: Plant concentrate B Dietary Supplement: Plant concentrate C Dietary Supplement: Plant concentrate D	-
NCT00882804	1	Parallel Assignment	Completed	Drug: Hemin infusion Drug: Placebo infusion	[193]
NCT00682370	1	Crossover Assignment	Completed	Drug: Saline solution Drug: Heme arginate	[194]
Non-registered	1	Parallel Assignment	Completed	Drug: Aspirin Drug: Sinvastin Drug: Sodium salt Drug: Lipoic acid	[195]
NCT02232308	1	Parallel Assignment	Completed	Drug: Nizatidine Drug: Lisinopril Drug: Placebo	-

^1^ NCT Number: ClinicalTrials.gov Identifier; ^2^ Not applicable.

**Table 2:** Clinical trials in which HO-1 expression is evaluated as a predictor of disease outcome.

Organ	ID ^1^	Disease	Phase	Intervention Model	Status	Interventions	Ref.
Liver	NCT04195438	Liver cancer	N/A ^2^	Single Group Assignment	Recruiting	Procedure: CO Testing Pre/Post Hepatic Resection Diagnostic Test: ABG Testing Pre/Post Hepatic Resection Diagnostic Test: CT Evaluations	-
	NCT00842205	HCV Nonalcoholic Steatohepatitis	data	Parallel Assignment	Unknown	Drug: Pegylated interferon Drug: Ribavarin	[201]
	NCT01112033 ^3^	HCV	N/A ^2^	N/A ^2^	Completed	None	[202]
Intestine/ colon	NCT03494764	Colitis, ulcerative	2	Crossover Assignment	Active, not recruiting	Hyperbaric Oxygen Therapy	-
	NCT02264496	Colorectal cancer	N/A ^2^	Parallel Assignment	Completed	Exercise	[203]

^1^ NCT Number: ClinicalTrials.gov Identifier; ^2^ Not applicable; ^3^ Case-control study.

**Table 3:** Clinical trials in which CO and Hemin have been used as treatment.

Treatment-Based	Organ	ID ^1^	Disease	Phase	Intervention Model	Status	Interventions	Ref.
CO	Intestine/ colon	NCT01050712 ^2^	Post-operative ileus	2	Parallel Assignment	Terminated	Drug: Inhaled Carbon Monoxide Drug: Synthetic Air	--
	Pancreas	NCT02567240	Chronic pancreatitis	2	Parallel Assignment	Completed	Carbon monoxide-bubbled mediums	[204]
Hemin	Stomach	NCT01206582	Gastroparesis	2	Parallel Assignment	Completed	Drug: Hemin Drug: Albumin	[102]
	Pancreas	NCT01855841	Acute pancreatitis	2	Parallel Assignment	Recruiting	Drug: Hemin Drug: Placebo	-

^1^ NCT Number: ClinicalTrials.gov Identifier; ^2^ 0 patients were enrolled.
